# Establishment of the Ivermectin Research for Malaria Elimination Network: updating the research agenda

**DOI:** 10.1186/s12936-015-0691-6

**Published:** 2015-06-11

**Authors:** Carlos J Chaccour, N Regina Rabinovich, Hannah Slater, Sara E Canavati, Teun Bousema, Marcus Lacerda, Feiko ter Kuile, Chris Drakeley, Quique Bassat, Brian D Foy, Kevin Kobylinski

**Affiliations:** Department of Internal Medicine, Clinica Universidad de Navarra, Pamplona, Spain; ISGlobal, Barcelona Ctr. Int. Health Res. (CRESIB), Hospital Clínic - Universitat de Barcelona, Barcelona, Spain; Instituto de Salud Tropical, Universidad de Navarra, Pamplona, Spain; Harvard T.H. Chan School of Public Health, Boston, MA USA; Department of Infectious Disease Epidemiology, MRC Centre for Outbreak Analysis and Modelling, Imperial College London, London, UK; Department of Clinical Tropical Medicine, Faculty of Tropical Medicine, Mahidol University, Bangkok, Thailand; Radboud University Medical Center, Nijmegen, The Netherlands; Fundação de Medicina Tropical Dr. Heitor Vieira Dourado/FIOCRUZ, Manaus, Brazil; Liverpool School of Tropical Medicine, Liverpool, UK; Malaria Centre, London School of Tropical Medicine and Hygiene, London, UK; Centro de Investigação em Saúde de Manhiça (CISM), Maputo, Mozambique; Department of Microbiology, Arthropod-borne and Infectious Diseases Laboratory, Immunology and Pathology, Colorado State University, Fort Collins, CO USA; Department of Entomology, Armed Forces Research Institute of Medical Sciences, Bangkok, Thailand; Entomology Branch, Walter Reed Institute of Research, Silver Spring, MD USA

**Keywords:** Ivermectin, Endectocides, Vector control, Residual malaria transmission, Malaria elimination, Research agenda

## Abstract

The potential use of ivermectin as an additional vector control tool is receiving increased attention from the malaria elimination community, driven by the increased importance of outdoor/residual malaria transmission and the threat of insecticide resistance where vector tools have been scaled-up. This report summarizes the emerging evidence presented at a side meeting on “Ivermectin for malaria elimination: current status and future directions” at the annual meeting of the American Society of Tropical Medicine and Hygiene in New Orleans on November 4, 2014. One outcome was the creation of the “Ivermectin Research for Malaria Elimination Network” whose main goal is to establish a common research agenda to generate the evidence base on whether ivermectin-based strategies should be added to the emerging arsenal to interrupt malaria transmission.

## Background

Malaria control efforts over the past 15 years have focused on the scale-up of long-lasting insecticide-treated nets (LLINs), indoor residual spraying with insecticides (IRS), and malaria diagnosis and treatment, and this package has succeeded in reducing malaria infections, morbidity and mortality [1]. Following the documented impact of increased malaria control, the concept of eradication was raised again in 2007 when Bill and Melinda Gates challenged the community to define long terms goals [2]. The concept of global eradication generated vigorous debate, but has increased political will and investments at a global level.

Vector control has played a central role in combating malaria and these advances are partly the consequence of a remarkable scale up in the distribution of LLINs and to a lesser extent to the use of IRS. As the selection pressure on vector populations mounts, insecticide resistance to the four classes of public health insecticides has risen worldwide [1,3,4]. In addition, malaria vector behaviour can shift from primarily biting indoors to outdoors and outside the time window that people are protected by LLINs or IRS [5], which was already a concern regarding Greater Mekong Subregion (GMS) vectors. The importance of residual malaria transmission, defined as the transmission that occurs in the temporal and spatial gaps left after deployment of our current core interventions (i.e. LLINs and IRS) highlights the need for new paradigms in vector control. There is also increasing need to target groups in which the core interventions are difficult to implement such as mobile populations and migrant workers [6].

Ivermectin is an endectocide drug, commonly used to treat neglected tropical diseases (NTDs), such as onchocerciasis, lymphatic filariasis and strongyloidiasis. It is also effective against scabies and lice [7,8]. Moreover, it reduces the survival of *Anopheles* mosquitoes that feed on an ivermectin treated person after a single standard oral dose [9,10]. Ivermectin mass drug administration (MDA) to humans has been theorized to be a potential malaria intervention, if it can be delivered in a manner or a formulation to extend its impact long enough to help suppress transmission in combination with current core interventions. Its ability to make human blood meals toxic to the mosquito offers the potential to directly target temporal and spatial transmission gaps regardless of vector feeding location or time. A relatively small ivermectin concentration will be sufficient to kill the mosquito before the parasite completes the ten day sporogonic cycle and the mosquito becomes infectious [9,10]. As such, ivermectin MDA could be a complementary measure to address residual malaria transmission, and appears as an extremely interesting addition to the current anti-malarial arsenal.

More than a quarter of all papers published about ivermectin as a malaria control tool were published in the last year [11-17]. Also, according to the MESA Track, a freely available database of all current research projects focusing on malaria elimination, provided by the Malaria Eradication Scientific Alliance (MESA), five out of nine identifiable grants on the subject are still ongoing at the beginning of 2015 [18] and the available body of evidence is expected to grow.

The meeting “Ivermectin for malaria elimination: current status and future directions” took place during the 2014 American Society of Tropical Medicine and Hygiene annual meeting. Participants included scientists representing some of the leading global health research institutions and representatives from funding agencies.

The main objectives were to discuss the most recent evidence of ivermectin MDA as a vector control tool as well as to promote the initiation of a joint collaborative effort to examine whether ivermectin-based strategies should be further developed. A number of issues were identified as requiring integration into the research framework including: the potential of ivermectin MDA to simultaneously impact malaria and multiple NTDs; safety concerns related to ivermectin treatment of persons with *Loa loa* co-infection [19], the monitoring protocols for SAEs post MDA [20]; and the urgent need for new tools to aid artemisinin resistance containment efforts in the GMS.

### Update of the existing research agenda

The meeting also provided the momentum to update the ivermectin for malaria research agenda, published in 2013 [21]. In that document, seven areas of knowledge gaps were identified, and several studies were proposed to address them. As recent publications have added additional information, an update to the agenda by categories is provided here.

### Human plasma levels and mosquito mortality

Ivermectin is extremely lipophilic, it is found in higher concentrations in dermal and adipose tissue than venous plasma [22], the resulting tissular concentration gradient may lead to higher concentrations in capillary than venous blood. This may be relevant as mosquitoes imbibe blood from subdermal capillaries and thus may ingest higher concentrations of ivermectin than would be predicted from determining the drug concentration in venous samples. Circulation of drugs at higher concentrations in capillary than venous blood has already been observed for other drugs, such as piperaquine [23,24]. An ongoing clinical trial will address whether capillary and venous blood impart different mosquito-lethal effects by performing both membrane and direct skin feeding assays at the same time [Feiko ter Kuile personal communication]. Importantly, it was recently demonstrated that the ivermectin mosquito-lethality lasts longer in females than males and in volunteers with a higher body mass index, possibly due to a higher body fat proportion [14], which could potentially imply differential dosage based on gender or expected body fat.

### Confirmation of lethal effects across a range of vector bionomics

Since 2013, new vectors have been characterized for their susceptibility to ivermectin including: *Anopheles funestus*, a primary vector in Africa [14], *Anopheles dirus*, *Anopheles minimus*, *Anopheles campestris*, and *Anopheles sawadwongporni*, important primary and secondary vectors in the GMS [25], and *Anopheles culicifacies*, a primary malaria vector in Asia [26]. Additionally, there is ongoing work to characterize *Anopheles aquasalis* [Marcus Lacerda, personal communication] and *Anopheles darlingi* [Gissella Vasquez, personal communication], both important Latin American malaria vectors. To date, all *Anopheles* tested have been susceptible at concentrations of ivermectin commonly found in humans or animals after a single standard dose.

### The effects of current ivermectin MDA programmes on malaria transmission

Mosquito survival assessments done after annual ivermectin MDAs in Senegal demonstrated that IVM MDA reduces wild *An. gambiae* survivorship for up to six days and reduces the proportion of *P. falciparum* infection among caught mosquitoes for up to two weeks following MDA [27,28]. Recently published work by Alout *et al.* [11] provided new evidence on the survival, sporozoite rate and parity rate of wild *An. gambiae* captured after IVM MDA campaigns in Senegal, Liberia, and Burkina Faso. Following ivermectin MDAs, survival of *An. gambiae* was reduced by more than 33% for the next six days, the sporozoite rate was reduced by over 77% in the following two weeks, and a reduction in parity (i.e., population age structure) was also observed. This provides ample evidence that IVM MDA can reduce entomological indices of *Plasmodium* transmission; however, there is a knowledge gap in the expected clinical and public health impact that can be expected after IVM MDA.

### Mathematical modelling

Recent work by Slater *et al.* [16] highlighted the potential impact of combining artemisinin-combination therapy (ACT) and IVM in MDA campaigns. In the model, the addition of IVM is predicted to increase the impact of ACT MDA and reduce the time needed to interrupt transmission. Slater’s model allows for a range of scenarios to be explored, including the impact of long-lasting IVM formulations and the impact of IVM MDA across varied transmission settings, seasonality profiles and vector species. Current work also includes extending the model to track the entomological parameters measured in the field and validating the model against data collected by Alout *et al.* [11]. Results from this validation will be used to predict the optimal time to commence MDA and the optimal spacing between rounds.

### Anti-sporogony effects

Previous work using *in vitro* parasites showed that sub-lethal concentrations of IVM reduce sporogony of *P. falciparum* in *An. gambiae* [29]. The *in vivo* clinical trial conducted by Ouédraogo *et al.* found no effect in naturally infected humans treated with ivermectin but this study was not powered to examine this and may have been confounded by the use of ACT. Field studies suggest that the primary driver of the approximate two week drop in sporozoite rates following IVM MDA is an equally lasting shift in mosquito population age structure as measured by parity rates [11]. Modelling indicates that the primary driver of ivermectin MDA effect is caused by a reduction in mosquito survivorship with mosquito-sublethal sporontocidal effects only having a small impact on parasite transmission [16].

### Safety and formulation assessments

Ouédraogo *et al.* [14] showed it is safe to administer a single standard dose of IVM (200 μg/kg) in combination with artemether-lumefantrine and in two standard doses spaced 48 hours apart, both supportive of a pathway towards implementation of IVM MDA within a malaria elimination strategy. Artemether-lumefantrine is an effective frontline anti-malarial drug in Africa, however, the six dose requirement raises the challenge of adherence with the full course for MDA. Slater’s model indicates that there is minimal additional transmission suppression effect to adding ivermectin during individual malaria treatment but the addition of ivermectin MDA to anti-malarial MDA could accelerate time to elimination and allow for elimination in settings where anti-malarial MDA alone would not achieve it [16]. The next step, being undertaken by ter Kuile and collaborators, is to determine the safety, tolerability, and potential drug-drug interaction of ivermectin with dihydroartemisinin-piperaquine, a more appropriate ACT for MDA given the longer half-life of the partner drug and three dose regimen. The same trial will explore safety and dose ranging of ivermectin applied on a daily basis for three days [18].

Recent animal model work [12] shows it is technically possible to safely sustain stable ivermectin blood concentrations at levels toxic to the mosquito by modifying the formulation to achieve prolonged sustained drug levels. Theoretically, this could be achieved by a number of existing technical and/or novel approaches. This becomes increasingly relevant, since increasing the duration of the mosquitocidal effect of the drug (i.e. time above mosquito-killing levels) is the parameter that has shown the greatest potential for impact in the model [16].

### Emerging resistance

Selection of *Anopheles* mosquitoes resistant to IVM to understand induction and mechanisms of resistance is ongoing in the Foy lab. First, mosquitoes respond to IVM exposure mainly by up-regulation of non-canonical transcripts. Second, older mosquitoes that have ingested a prior blood meal are more sensitive to a second IVM-containing blood meal than young mosquitoes ingesting their first blood meal containing IVM, this has important implication for possible IVM MDA since it is only the older *Anopheles* that can transmit the parasite. In addition, IVM-sensitive wild *An. gambiae* collected from the IVM MDA studies had a high prevalence of the *Kdr* 1014 F mutation associated with pyrethroid and DDT resistance, which suggests that potential ivermectin resistance is independent of pyrethroid resistance [Haoues Alout, personal communication].

The 2013 research agenda [21] proposed three studies to provide evidence in support of this new vector control strategy.

1. A placebo-controlled, cluster-randomized clinical trial of IVM MDA over a non-continuous malaria transmission season, proving the concept that a potentially deliverable IVM MDA scheme would result in reduced vectorial capacity and translate into parasitological and clinical endpoints.
2. An individual-randomized clinical trial where confirmed cases or asymptomatic carriers are randomized to ACT vs ACT + IVM treatment with entomological endpoints assessed through colonized *Anopheles*, proving the concept that adding IVM to an ACT regime further reduces the amount of mosquitoes that become infectious. Additional endpoints include safety and pharmacokinetic (PK) interaction assessments.
3. A placebo-controlled, cluster-randomized trial in which livestock receive endectocide treatment, with entomological, parasitological and clinical endpoints in the human population, answering the question whether targeting zoophagic vectors with treated livestock could result in reduced malaria transmission to humans.

Although none of the suggested trials has taken place in full yet, recent work has provided partial answers to the questions originally posed. Alout *et al.* [11] has confirmed reduced survivorship after IVM MDA in different transmission settings, one of the key questions of the first study proposed. Ouédraogo *et al.* [14] have proven IVM safe in two repeated doses and in combination with artemether-lumefantrine in *P. falciparum* carriers, with no significant PK interactions between the drugs. Ongoing work by ter Kuile and collaborators aims to determine the safety of IVM at higher doses and co-administration with dihydroartemisinin-piperaquine.

In summary, the two main key decision nodes that can be addressed by new data are: 1) the feasibility of using IVM MDA as an additional vector control tool while determining its effectiveness across different scenarios and the comparative costs with other new or existing interventions, and 2) the viability of the product development and regulatory pathways. Funding should be prioritized to help make these key decisions.

A key concept is the potential impact of the current oral formulation and standard dose of 150–200 mcg/kg on transmission dynamics. Modelling clearly shows that the time the drug remains in human blood above mosquito-killing levels is a key determinant of the epidemiological outcome of IVM MDA. There are different ways to increase the time above the target concentration: different formulations, higher or repeated doses, and PK enhancement by blocking the P-glycoprotein or metabolic pathways. Although the current formulation in repeated doses can be used to prove the concept, scalability is a concern and multiple rounds of MDA in the field might only be practical for short rainy seasons or defined outbreaks. Data addressing the minimum time above the target concentration required to interrupt transmission is needed to answer this question appropriately and chose a suitable formulation or dosage scheme into which further investment should be made.

### The regulatory pathway

Ivermectin lies outside the classical definition of a transmission blocking drug as it does not impart a gametocytocidal effect, and its sporontocidal effects appear modest by the minimum essential requirements for transmission blocking drug standards set forth by the Medicines for Malaria Venture [30]. Classical transmission blocking drugs (e.g. primaquine) prevent onwards transmission from the treated individual to the mosquito population, but this means that there might be a delay before any effect is seen in terms of transmission from mosquito-to-human. However, ivermectin’s mosquito lethal and sublethal effects can quickly suppress the infectious vector population following MDA and thus immediately reduce mosquito-to-human transmission.

In spite of its excellent safety profile [31], IVM does not kill asexual stage parasites at human relevant concentrations, nor does it offer prophylaxis against newly infecting malaria parasites. This is an issue faced by other transmission blocking interventions such as primaquine [32] or vaccines [33]. However, there are direct personal health benefits to taking IVM if one considers the numerous NTD parasites that often afflict people in malaria-endemic communities [13,34].

Two recent developments show promise to ease the pathway for malaria transmission blocking interventions. One is the WHO recommendation to implement low dose primaquine as a *P002E falciparum* gametocytocide in elimination areas and where artemisinin resistance constitutes a threat, without the need to test for G6PD deficiency, even in the absence of direct personal clinical benefits [32]. The second is the advances driven by the PATH Malaria Vaccine Initiative in framing the regulatory pathway for a transmission blocking vaccine [33].

There may be strategies, depending on dose and formulation, for targeting multiple diseases simultaneously. This possibility, along with potential safety and efficacy issues should be considered in Target Product Profile (TPP) and development/regulatory pathway proposals. These unique and complex facets of ivermectin MDA make the development and regulatory pathways for novel ivermectin applications or formulations for malaria elimination difficult to address as it requires oversight from both drug and vector control experts and regulatory agencies. Collaboration between the WHO Global Malaria Program and Neglected Tropical Diseases Program, with input from product development and regulatory experts, would be critical to decreasing perceived clinical and regulatory barriers.

A framework addressing the potential issues for the development of ivermectin for malaria control is proposed here (Table 1).

**Table 1:** **Framework for the evaluation of IVM as a potential malaria elimination tool**

**Criteria**	**Background**	**Examples of questions to be answered**
**Efficacy**	Robust empirical data coupled with modelling will be needed to support a specific target efficacy.	What epidemiological impact can be expected at different levels of mosquito survival/EIR in different endemicities?
	For early development, the proposed biological surrogate for effectiveness is prevention of mosquitoes (both colonized and wild) to become infectious as measured by the proportion of mosquitoes which survive through the sporogonic cycle and sporozoite prevalence in those surviving.	Are lab and semi-field assays acceptable alternatives to field studies, and which assay(s) should be used to determine the above endpoints? (e.g. SMFA, DMFA, DFA, wild mosquito survival and EIR)
		How long should the transmission suppression effect last?
	The ultimate goal of reducing entomological endpoints (i.e. the sporozoite rate and the EIR) and human endpoints (the molFOI and incidence of clinical episodes in the selected strata) must be considered for proposed MDA trials.	Should this be tested as a stand-alone intervention or in combination with other transmission-blocking interventions? (i.e. primaquine, ACT)
		How many encounters are needed to deliver the optimal intervention? (single encounter vs multiple)
	The suggested TPP for TBV uses >85% transmission–blocking efficacy as a target [33]. This number however, should be interpreted carefully regarding ivermectin because the primary effect of ivermectin is to kill the vector, not to block transmission.	Which parasitological/human endpoints should be assessed in phase III trials?
		Can a cluster randomized trial provide enough power to prove efficacy using human endpoints?
**Dose**	This criterion is closely related to efficacy as there is a correlation between plasma levels and mosquito mortality [14].	What target plasma levels should oral dose regimes or slow release formulations have?
	The duration of effective mosquito-lethal plasma concentrations can be increased using higher dose or with novel slow release formulations.	Should target concentrations be determined in plasma or whole blood? Should the samples be obtained from venous or capillary blood, or the midgut of freshly fed mosquitoes? What limits of detection should be sought in methods to assess systemic levels?
	The maximum dose administered safely to healthy volunteers was 2 mg/kg [36].	Is there a role for differential dosing based on gender, BMI or total body fat?
**MDA coverage**	Robust empirical data coupled with modelling will be needed to support a specific coverage target.	Is there added value in increasing coverage beyond 80% of the population?
	Current MDA for onchocerciasis control use 80% of total population as target.	Is it safe in children <15 kg and pregnant/breast feeding women?
	Current use of IVM excludes children <15 kg or <90 cm, as well as pregnant and breast feeding women unless their risk for LF or onchocerciasis is high.	What is the safety profile of IVM in pregnancy and breast feeding mothers?
**Route of administration/ Presentation**	There is extensive experience in MDA using the oral formulation.	What non-parenteral slow release formulations could be used?
	Injectable formulations are not desired for human MDA.	
**Safety**	Current MDA programmes for onchocerciasis control report no severe adverse reactions and their rate of moderate adverse reactions is ≤ 1.3% [31].	Would higher or more frequent doses translate into a higher adverse event rate?
	An adverse event rate less than 1:10.000 is the referenced used in the development of new anti-malarial drugs [20].	Is there any local toxicity to be taken into account in the development of new formulations?
**Drug-Drug interactions**	Ivermectin is metabolized by the cytochrome P4503A4 and excreted by the P-gp [37,38].	Is there any relevant interaction of ivermectin with anti-malarial, anti-retroviral or TBC medicines?
	Co-administration with Artemeter-lumefantrine has been found safe and did not alter lumefantrine concentrations [14].	
	IVM is commonly co-administered with other anti-helmintics such as albendazole [13].	Are these risks manageable?
	IVM for onchocerciasis is commonly used in areas with high HIV and TBC prevalence.	
**Spectrum**	Different *Anopheles* species, even in the same species complex, have different sensitivity to IVM. (i.e. different LC_50_).	How would differing IVM susceptibilities of various primary malaria vectors in the same region alter efficacy of ivermectin MDA?
	Any scheme should target the main vectors of areas selected for elimination.	
**Disease targets**	The effects of IVM on co-endemic NTDs offers direct personal benefit to those treated and which may increase and advantages regarding community acceptance and compliance [34].	What additional benefits on NTDs and ectoparasites can be expected from a wider use of IVM targeting malaria?
		Should the design of a new product target malaria and only have beneficial non-target side effects against NTDs or should malaria and NTDs be targeted from the beginning?
**Projected stability**	Current recommended storage conditions are <30°C.	What is the stability of any potential new formulation?
	Ideal stability is > 60 months in hot/humid climates [20].	

ACT: artemisinin combination therapy, BMI: body mass index, DFA: direct feeding assay, DMFA: direct membrane feeding assay, EIR: entomological inoculation rate, HIV: human immunodeficiency virus, IVM: ivermectin, LC_50_: lethal concentration that kills 50% of feeding mosquitoes, MDA: mass drug administration, molFOI: molecular force of infection, NTD: neglected tropical diseases, P-gp: P-glycoprotein, SMFA: standard membrane feeding assay, TBC: tuberculosis, TBV: transmission blocking vaccine, TPP: target product profile.

Three options for the use of IVM for malaria elimination should be evaluated:

1. Mass drug administration with IVM in addition to core interventions already in place: repeat doses or single dose long-lasting formulations for the whole population during the rainy season to target the vector population, and in particular the infectious vector population to reduce clinical incidence.
2. Targeted population strategy: repeat doses or single long-lasting dose to at-risk individuals, or specific groups that constitute a major reservoir of infection in a population, in addition to treatment with an ACT. For example, adult male forest workers in the GMS typically have higher infection rates than the general population as they have more exposure to the forest-dwelling malaria vector, *Anopheles dirus*.
3. Mass drug administration with ACT and current or novel long-lasting IVM formulations: combining these drugs in areas aiming to eliminate malaria. This would target transmission at the parasite and vector levels. IVM also has the potential to reduce resistance selection pressure against ACT by minimizing the number of sporozoite challenges following drug treatment.

### Formation of the Ivermectin Research for Malaria Elimination Network (IVERMEN)

Following the positive interaction among interested parties the “Ivermectin Research for Malaria Elimination Network”, was formed with interested partners from academia, the private sector, non-government organizations and funding agencies. The goals of the Network are:

- Convene stakeholders from the malaria and NTD communities to facilitate access to: current and novel ivermectin formulations, field sites, and protocols to standardize impact assessment methodologies.
- Harmonize ivermectin for malaria transmission suppression related research interests across the diverse landscape of researchers and transmission settings world-wide.
- Work to further improve the common Research Agenda and framework for potential development of this tool

Members of the global health community interested in the field are encouraged to join the working group by emailing mail@ivermen.org. Current members, upcoming events and supportive documents are listed in the IVERMEN website [35].

## Abbreviations

ACT: Artemisinin Combination Therapy
GMS: Greater Mekong Subregion
ILLNS: Long-Lasting Insecticide-treated Nets
IVERMEN: Ivermectin Research for malaria Elimination Network
IRS: Indoor Residual Spraying
IVM: Ivermectin
MDA: Mass Drug Administration
MESA: Malaria Eradication Scientific Alliance
NTDs: Neglected Tropical Diseases
RMT: Residual Malaria Transmission
SAE: Severe Adverse Events
WHO: World Health Organization
